# Systematic Review of the Effects of Iron on Cardiovascular, Kidney, and Safety Outcomes in Patients With CKD

**DOI:** 10.1016/j.ekir.2025.01.029

**Published:** 2025-01-29

**Authors:** Bernard Chan, Amanda Varghese, Sunil V. Badve, Roberto Pecoits-Filho, Murilo Guedes, Clare Arnott, Rebecca Kozor, Emma O’Lone, Min Jun, Sradha Kotwal, Geoffrey A. Block, Glenn M. Chertow, Scott D. Solomon, Muthiah Vaduganathan, Vlado Perkovic, Brendon L. Neuen

**Affiliations:** 1 Department of Cardiology, Royal North Shore Hospital, Sydney, New South Wales, Australia; 2 Faculty of Medicine and Health, University of Sydney, Sydney, New South Wales, Australia; 3 Department of Renal Medicine, Royal North Shore Hospital, Sydney, New South Wales, Australia; 4 Renal and Metabolic Division, The George Institute for Global Health, University of New South Wales, Sydney, New South Wales, Australia; 5 Department of Renal Medicine, St George Hospital, Sydney, New South Wales, Australia; 6 Arbor Research Collaborative for Health, Ann Arbor, Michigan, USA; 7 Department of Cardiology, Royal Prince Alfred Hospital, Sydney, New South Wales, Australia; 8 Department of Nephrology, Prince of Wales Hospital, Sydney, New South Wales, Australia; 9 US Renal Care, Denver, Colorado, USA; 10 Department of Medicine, Stanford University School of Medicine, Stanford, California, USA; 11 Department of Epidemiology and Population Health, Stanford University School of Medicine, Stanford, California, USA; 12 Department of Health Policy, Stanford University School of Medicine, Stanford, California, USA; 13 Cardiovascular Division, Brigham and Women’s Hospital, Harvard Medical School, Boston, Massachusetts, USA

**Keywords:** cardiovascular outcomes, chronic kidney disease, heart failure, iron therapies

## Abstract

**Introduction:**

Heart failure and chronic kidney disease (CKD) are closely associated, and iron deficiency is highly prevalent in both conditions. However, major cardiovascular and nephrology guidelines offer contrasting recommendations for iron use. We evaluated the effects of iron versus usual care or placebo on the clinical outcomes in patients with CKD.

**Methods:**

We conducted a systematic review and meta-analysis of randomized trials on i.v. or oral iron in patients with CKD (PROSPERO CRD42023453468). We searched Medline, Embase, and the Cochrane Register from database inception until February 1, 2024 to identify eligible trials. We determined the overall results and stratified them by dialysis- and nondialysis-requiring CKD using random effects models, with certainty of evidence assessed using the Grading of Recommendations Assessment, Development, and Evaluation approach. The primary composite endpoint was hospitalization for heart failure or cardiovascular death.

**Results:**

We identified 45 trials that met the inclusion criteria. Compared with usual care or placebo, iron reduced the risk of the primary composite endpoint (1659 events; risk ratio [RR]: 0.84, 95% confidence interval [CI]: 0.75–0.94; moderate certainty), an effect consistent across dialysis and nondialysis requiring CKD (*P*-heterogeneity = 0.70). The effect on the primary endpoint appeared driven by both components of hospitalization for heart failure (RR: 0.77; 95% CI: 0.61–0.96; moderate certainty) and cardiovascular death (RR: 0.81; 95% CI: 0.65–1.02; low certainty). The incidence of serious adverse events was lower for iron compared with usual care or placebo (RR: 0.90, 95% CI: 0.82–0.98; moderate certainty; *P*-heterogeneity = 0.09).

**Conclusion:**

Iron therapy may reduce the risk of heart failure and cardiovascular death in patients with CKD. Randomized trials evaluating the effects of iron on clinical outcomes are needed, especially in nondialysis patients with CKD with or without anemia.

## Graphical abstract

**Figure:**
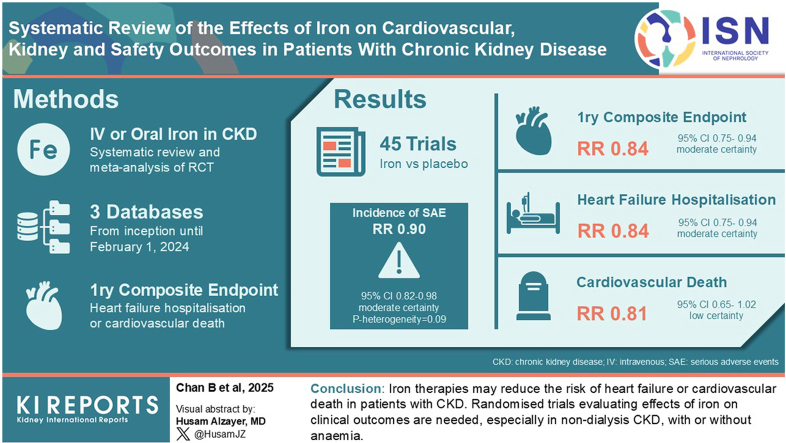

Iron is an essential trace element with biological roles in hemoglobin synthesis, cellular function, and oxygen transport. Approximately one-third of patients with CKD have relative or absolute iron deficiency,^1^ both of which are associated with a reduced health-related quality of life, cardiovascular events, and death, independent of anemia.^2^^,^^3^ In patients with advanced CKD, iron deficiency and reduced erythropoietin synthesis are key drivers of anemia. Among patients with kidney failure, iron deficiency and anemia are virtually ubiquitous because of the effects of blood loss and chronic inflammation; chronic inflammation leads to upregulation of hepcidin and diminished intestinal iron absorption as well as sequestration of iron in reticuloendothelial cells.^4^ Therefore, in patients with dialysis- and nondialysis-requiring CKD, iron is frequently administered to manage anemia and reduce the need for and/or dosing of erythropoiesis stimulating agents (ESAs).

In patients with heart failure with reduced ejection fraction who are iron deficient, multiple randomized, placebo-controlled trials have indicated that i.v. iron administration reduces hospitalization for heart failure and improves functional status and quality of life.^5^ As a result, clinical practice guidelines from the European Society of Cardiology and other expert scientific statements recommend i.v. iron to improve functional status and clinical outcomes in patients with heart failure with reduced ejection fraction independent of anemia.^6^ In contrast, kidney disease–focused guideline development groups, including Kidney Disease Improving Global Outcomes, recommend screening for iron deficiency only when anemia develops, and largely focus on iron therapies as a tool to manage anemia, with CKD trials mostly evaluating effects of iron on hemoglobin concentrations and ESA dosing.^7^

Inconsistent recommendations from widely accepted specialty care guidelines are noteworthy because heart failure and CKD frequently coexist, and the presence of one complicates the management of the other.^8^^,^^9^ The bidirectional relationship between these 2 conditions suggests that iron might reduce the risk of heart failure and/or other cardiovascular events in patients with CKD. In addition to trials on heart failure that included many patients with CKD, several randomized trials of iron therapies in primary CKD populations have been conducted comparing iron versus placebo or usual care, newer versus older generation formulations, i.v. versus oral supplementation, and higher versus lower doses of oral iron. To date, these results have not been systematically evaluated or quantitatively synthesized.

Therefore, we conducted a systematic review and meta-analysis to evaluate the effects of iron therapy on the cardiovascular, renal, and safety outcomes in patients with dialysis- and nondialysis requiring CKD.

## Methods

We conducted and reported this systematic review and meta-analysis in accordance with the Preferred Reporting Items for Systematic reviews and Meta-Analyses statement, with prospective registration on PROSPERO (CRD42023453468).

### Search Strategy and Selection Criteria

We searched Medline, Embase, and the Cochrane Central Register of Controlled Trials (CENTRAL) from database inception to February 1, 2024 using search terms, including “iron therapies” and related phrases, the names of individual iron compounds, “chronic kidney disease,” “heart failure,” and terms related to randomized controlled trials. The full search strategy, including text words and medical subject headings, is provided in Supplementary Table S1).

All randomized controlled trials of adults (aged ≥ 18 years) with CKD comparing the effects of i.v. or oral iron with usual care or placebo on cardiovascular, kidney, and safety outcomes were eligible for inclusion. CKD was defined as an estimated glomerular filtration rate < 60 ml/min per 1.73 m^2^ or an albumin-to-creatinine ratio > 30 mg/g. Because the majority of patients with dialysis-requiring CKD receive regular iron administration as part of their usual care,^10^ we included trials that evaluated different dosing strategies (e.g,. proactive vs. reactive iron administration) in this population. We also included trials comparing iron-based phosphate binders (i.e., sucroferric oxyhydroxide and ferric citrate coordination complex) with usual care or a placebo. Secondary aims included evaluating the cardiovascular, kidney, and safety outcomes reported from trials that assessed the effects of the following: (i) newer versus older iron formulations, (ii) i.v. versus oral iron, and (iii) higher versus lower doses of oral iron (outside of dialysis-requiring CKD), where data were available. Multiarm trials were included by combining 2 or more arms receiving iron therapies (when the comparison arm was usual care or placebo) or by combining treatment arms based on the generation or dose of the iron therapy (when there was no usual care or placebo arm). Trials that focused on adults who underwent kidney transplantation were included.

### Data Extraction

Two authors (BC and AV) independently screened the titles and abstracts of all identified articles and, when indicated, reviewed the full-text reports to identify potentially relevant studies. Any disagreements regarding the eligibility of the studies were discussed and resolved by a third author (BLN). The same 2 authors (BC and AV) independently extracted all data using Covidence systematic review software 2023 (Veritas Health Innovation, Melbourne, Australia) and assessed the risk of bias at the study level using the Cochrane Risk of Bias 1 tool. In addition, we contacted the investigators to request additional unpublished trial data on the key outcomes. Any discrepancies in the data extraction were resolved by the third author (BLN).

### Outcomes

The primary endpoint was a composite of heart failure hospitalization and cardiovascular death. Other cardiovascular outcomes included hospitalization for heart failure, cardiovascular death, myocardial infarction (MI), and stroke. Where data were available, we evaluated the effects of iron therapies on kidney outcomes, including changes in estimated glomerular filtration rate, proteinuria, and kidney failure requiring dialysis. In addition, we assessed the incidence of all-cause mortality and serious adverse events.

### Data Analysis and Synthesis

We prespecified that treatment effects on clinical outcomes were to be quantitatively synthesized using a random effects model to obtain summary treatment effect estimates expressed as relative risks with associated 95% CIs. Our preference was to use hazard ratios from time-to-first-event analyses; however, where these were unavailable, we used the estimated treatment ratios effects obtained from recurrent event analyses. For treatment comparisons in which only the number of events and participants were reported, we calculated and pooled the risk to maximize the information obtained from the trial-level data.

We summarized the effects on estimated glomerular filtration rate and proteinuria without conducting inference tests because of substantial heterogeneity in how data on these outcomes were reported (some trials reported values at baseline and follow-up, whereas others reported changes from baseline in each treatment arm), as well as imbalances in baseline values between the active and control arms.

Because most patients with dialysis-requiring CKD are routinely administered iron to support erythropoiesis,^10^ we conducted stratified analyses according to dialysis- and nondialysis-requiring CKD subgroups. Between-study variation was evaluated based on *P*-heterogeneity values obtained from a random-effects model, with standard chi-square tests for heterogeneity used to assess differences between dialysis- and nondialysis-requiring subgroups.

We summarized the certainty of evidence for each outcome using the Grading of Recommendations Assessment, Development, and Evaluation approach based on the following domains: within-study risk of bias, indirectness of evidence, unexplained heterogeneity or inconsistency of results, and imprecision of results.^11^ Statistical analyses were performed using STATA version 17.0 (StataCorp, 2023).

## Results

Our search strategy yielded 2317 records, of which 248 were assessed for eligibility (Supplementary Figure S1). We identified 45 trials that met the inclusion criteria. Overall, 18 and 26 trials enrolled participants with dialysis- and nondialysis-requiring CKD, respectively; and 1 trial enrolled participants from both groups. Five trials studied participants who had heart failure with reduced ejection fraction, where treatment effects in CKD subgroups were reported.^12^, ^13^, ^14^, ^15^, ^16^ There were 26 trials that compared the effects of iron versus placebo or usual care,^12^, ^13^, ^14^, ^15^, ^16^, ^17^, ^18^, ^19^, ^20^, ^21^, ^22^, ^23^, ^24^, ^25^, ^26^, ^27^, ^28^, ^29^, ^30^, ^31^, ^32^, ^33^, ^34^, ^35^, ^36^, ^37^8 trials of i.v. versus oral iron,^38^, ^39^, ^40^, ^41^, ^42^, ^43^, ^44^, ^45^ 9 trials of newer versus older iron formulations,^46^, ^47^, ^48^, ^49^, ^50^, ^51^, ^52^, ^53^, ^54^ and 2 trials of higher versus lower oral iron doses.^55^^,^^56^ Ten trials assessed the effects on a primary clinical endpoint, whereas biochemical or surrogate outcomes were the primary focus in 35 trials. Details of trials comparing iron versus usual care or placebo are summarized in Table 1, whereas trials comparing other treatments are summarized in Supplementary Tables S2 to S4.

**Table 1:** Characteristics of trials comparing iron therapies vs. usual care or placebo

Trial	Year	Participants with CKD/total study population	Duration of follow-up	Primary population	Intervention	Comparator	Mean age, yrs	Female (%)	Primary outcome
Besarab *et al.*^18^	2000	42/42	6 mo	Hemodialysis	i.v. iron dextran (target TSAT > 30%)	i.v. iron dextran (target TSAT: 20%–30%)	60.8	40.5	ESA dose needed to maintain Hb levels
DRIVE^23^	2007	134/134	6 wks	Hemodialysis	i.v. ferric gluconate	Usual care	59.9	49.6	Change in Hb
FAIR-HF^12^	2009	203/459	24 wks	HFrEF (NYHA II with LVEF ≤ 40% or NYHA III with LVEF ≤ 45%)	i.v. ferric carboxymaltose	i.v. saline	67.7	53.2	Self-reported PGA and NYHA functional class
McIntyre *et al.*^31^	2009	63/63	5 wks	Hemodialysis	p.o.fermagate	p.o. placebo	59.1	28.6	Change in serum phosphate
Charytan *et al.*^22^	2013	97/513	30 d	Hemodialysis	i.v. ferric carboxymaltose	Usual care	55.9	33.0	Incidence in treatment-emergent adverse events
		416/513		Nondialysis CKD			64.5	67.3	
Yokoyama *et al.*^36^	2014	90/90	8 wks	Nondialysis CKD	p.o. ferric citrate	p.o. placebo	65.1	41.9	Change in serum phosphate
Block *et al.*^21^	2015	149/149	12 wks	Nondialysis CKD	p.o. ferric citrate	p.o. placebo	65.0	66.0	Change in TSAT and serum phosphate
CONFIRM-HF^16^	2015	105/304	52 wks	HFrEF ≤ 45%	i.v. ferric carboxymaltose	i.v. saline	69.2	46.8	Change in 6MWT distance
CRUISE 1 and 2^25^	2015	599/599	48 wks	Hemodialysis	i.v. ferric pyrophosphate citrate	i.v. standard dialysate	58.4	36.4	Mean change in Hb
PRIME^27^	2015	103/103	9 mos	Hemodialysis	i.v.ferric pyrophosphate citrate	i.v. standard dialysate	59.0	38.8	Change in ESA dose to maintain Hb 9.5–11.5g/dl
Floege *et al.*^26^	2015	644/644	52 wks	Hemodialysis or peritoneal dialysis	p.o. sucroferric oxyhydroxide	p.o. sevelamer	55.4	41.5	Change in serum phosphate
Lewis *et al.*^34^	2015	441/441	52 wks	Hemodialysis or peritoneal dialysis	p.o. ferric citrate	p.o. calcium acetate and/or sevelamer	54.5	38.8	Mean change in serum phosphate
Fishbane *et al.*^24^	2017	233/233	24 wks	Non-dialysis CKD	p.o. ferric citrate	p.o. placebo	65.4	63.1	Proportion of patients with Hb increase ≥1.0 g/dl
Koiwa *et al.*^29^	2017	213/213	12 wks	Hemodialysis	p.o. sucroferric oxyhydroxide	p.o. sevelamer	60.9	34.9	Change in serum phosphate
Iguchi *et al.*^28^	2018	40/40	12 wks	Nondialysis CKD	p.o. ferric citrate	Usual care	70.1	62.5	Change in serum ferritin, FGF23 and PTH
Block *et al.*^20^	2019	203/203	9 mos	Nondialysis CKD (eGFR ≤ 20 ml/min per 1.73 m^2^)	p.o. ferric citrate	Usual care	62.4	38.0	Change in Hb, TSAT, phosphate, FGF23 and PTH
PIVOTAL^30^	2019	2141/2141	2.1 yrs	Hemodialysis	i.v. iron sucrose (proactive regimen)	i.v.iron sucrose (reactive regimen)	62.8	34.7	Composite of nonfatal myocardial infarction, nonfatal stroke, heart failure hospitalization, or death
AFFIRM-AHF^15^	2020	580/1108	52 wks	HFrEF with LVEF < 50%	i.v. ferric carboxymaltose	i.v. placebo	74.5	47.2	Composite of heart failure hospitalization and cardiovascular death
van den Oever *et al.*^35^	2020	200/200	13 mos	Hemodialysis	i.v.iron sucrose	Usual care	68.9	31.4	Median percentage of Hb values in target range (6.8–7.4 mmol/l)
Susantitaphong *et al.*^33^	2020	200/200	6 mos	Hemodialysis	i.v.iron (ferritin target 600–700 ng/ml)	i.v. iron (ferritin target 200–400 ng/ml)	52.9	46.5	Effect on ESA dose (erythropoietin resistance index)
AEGIS-CKD^32^	2021	167/167	52 wks	Nondialysis CKD	p.o. ferric maltol	p.o. placebo	67.4	70.1	Change in Hb
Zununi Vahed *et al.*^37^	2021	60/60	6 mos	Hemodialysis	i.v. iron (high dose)	i.v. iron (low dose)	61.2	40.0	ESA dose needed to maintain Hb levels 10–12 g/dl
The Iron and the Heart Study^19^	2021	54/54	3 mos	Nondialysis CKD	i.v. ferric derisomaltose	i.v. saline	59.6	51.9	Change in 6MWT distance
IRONMAN^13^	2022	730/1137	2.7 yrs	HFrEF with LVEF ≤ 45%	i.v. ferric derisomaltose	Usual care	73.3	26.4	Composite of heart failure hospitalization and cardiovascular death
HEART-FID^14^	2023	1553/3065	12 mos	HFrEF with LVEF ≤ 40%	i.v.ferric carboxymaltose	i.v.placebo	68.6	33.8	Hierarchical composite of death and heart failure hospitalization, or change from baseline in 6MWT distance
MAINTAIN-IRON^17^	2023	79/79	12 mos	Hemodialysis	i.v. iron sucrose (high dose)	i.v. iron sucrose (low-dose)	70.8	55.7	ESA dose needed to maintain Hb of 10–12 g/dl

6MWT, 6-minute walk test; CKD, chronic kidney disease; eGFR, estimated glomerular filtration rate; ESA, erythropoiesis-stimulating agent; FGF23, fibroblast growth factor-23; Hb, hemoglobin; HFrEF, heart failure with reduced ejection fraction; LVEF, left ventricular ejection fraction; NHYA, New York Heart Association Class; PGA, patient global assessment; PTH, parathyroid hormone; TSAT, transferrin saturation.

The overall risk of bias was low in the larger event-driven trials in patients with dialysis-requiring CKD and heart failure with a reduced ejection fraction (Supplementary Table S5). However, there was substantial heterogeneity in the risk of bias across the other trials and comparisons (Supplementary Tables S5–S8).

**Figure 1:**
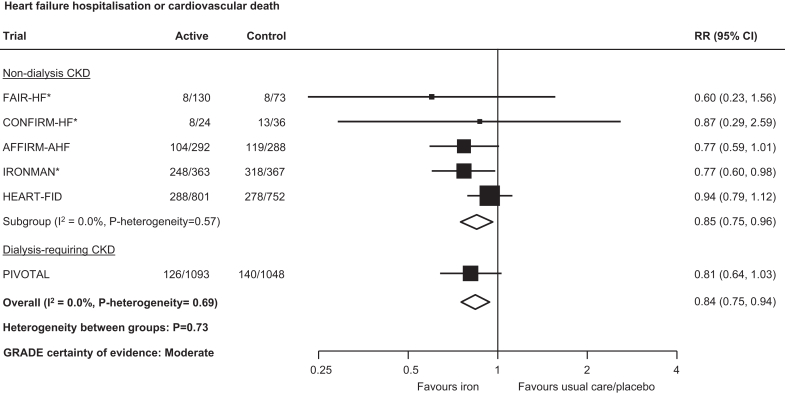
Effect of iron therapies versus usual care or placebo on hospitalization for heart failure or cardiovascular death in people with CKD. ∗First and recurrent hospitalizations for heart failure or cardiovascular death. CI, confidence interval; CKD, chronic kidney disease; GRADE, Grading of Recommendations, Assessment, Development and Evaluation; RR, relative risk.

**Figure 2:**
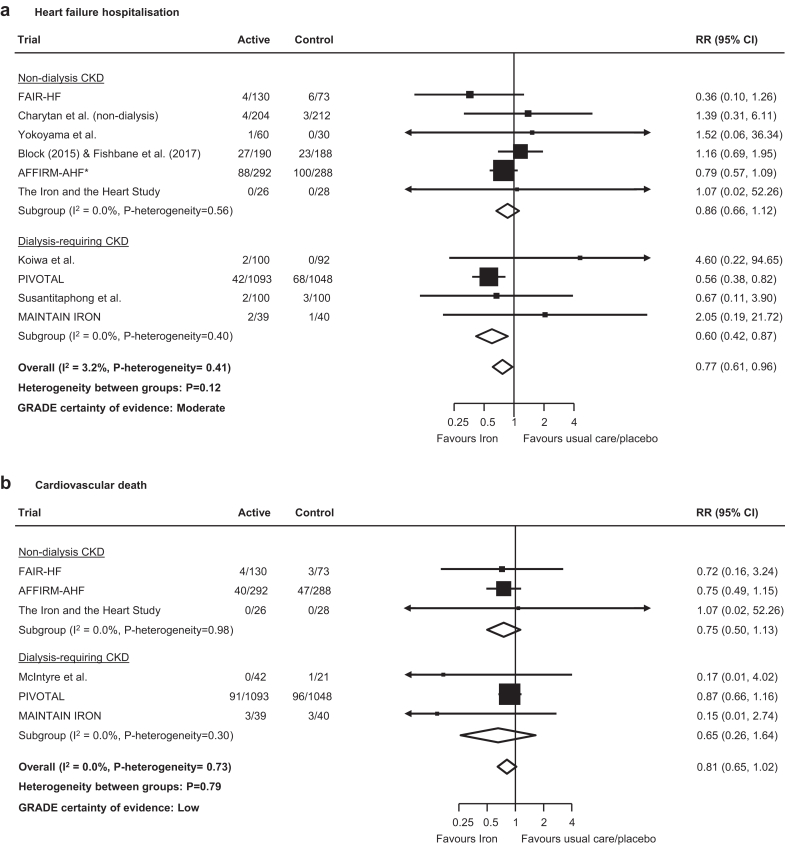
Effect of iron therapies on (a) hospitalization for heart failure and (b) cardiovascular death in people with CKD. ∗First and recurrent hospitalizations for heart failure. CI, confidence interval; CKD, chronic kidney disease; GRADE, Grading of Recommendations, Assessment, Development and Evaluation; RR, relative risk.

Overall, randomization to iron reduced the risk of hospitalization for heart failure or cardiovascular death by 16% (1658 events; RR: 0.84, 95% CI: 0.75–0.94; moderate certainty; Figure 1), an effect consistent across dialysis- and nondialysis-requiring CKD subgroups (RR: 0.85, 95% CI: 0.75–0.96 and RR: 0.81, 95% CI: 0.64–1.03; *P*-heterogeneity = 0.73). Risk reductions on the primary composite endpoint appeared driven by both components of hospitalization for heart failure (376 events; RR: 0.77; 95% CI: 0.61–0.96; moderate certainty; Figure 2) and cardiovascular death (288 events; RR: 0.81; 95% CI: 0.65–1.02; low certainty; Figure 2), with no evidence of heterogeneity across dialysis- and nondialysis-requiring CKD subgroups (both *P*-heterogeneity > 0.10). For i.v. versus oral iron and newer versus older iron formulations, no clear differences were observed for these outcomes, although data were limited to a small number of trials (Supplementary Figure S2–S3).

**Figure 3:**
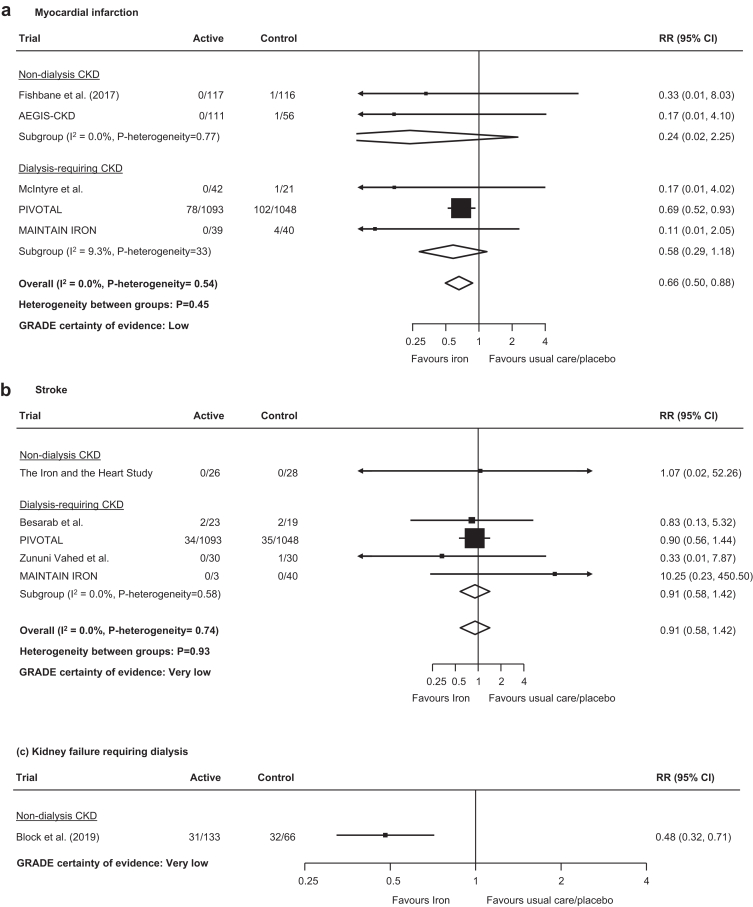
Effects of iron therapies versus usual care or placebo on (a) myocardial infarction, (b) stroke and (c) kidney failure in people with CKD. CI, confidence interval; CKD, chronic kidney disease; GRADE, Grading of Recommendations, Assessment, Development and Evaluation; RR, relative risk.

In contrast to hospitalizations for heart failure or cardiovascular death, fewer MIs and stroke events were recorded in the included trials. Randomization to iron reduced the risk of MI (187 events; RR: 0.66, 95% CI: 0.50–0.88; low certainty; Figure 3), driven entirely by the PIVOTAL trial in dialysis-requiring CKD, with few events in other trials. No clear effect on stroke was observed (74 events; RR: 0.91, 95% CI: 0.58–1.42; very low certainty; Figure 3). There were too few data to reliably assess effects on MI or stroke for i.v. versus oral iron and newer versus older iron formulations (Supplementary Figures S2–S3).

In patients with nondialysis-requiring CKD, randomization to ferric citrate coordination complex reduced the risk of kidney failure based on data from 1 trial (63 events; RR: 0.48, 95% CI: 0.32–0.71; very low certainty; Figure 3). Generally, no effects on estimated glomerular filtration rate or proteinuria were observed with iron versus usual care or placebo; however, heterogeneity in data presentation and imbalances between treatment arms precluded quantitative synthesis (Supplementary Tables S9 and S12). For other comparisons, there were few reports on kidney outcomes (Supplementary Figure S2 and Tables S10–S16).

**Figure 4:**
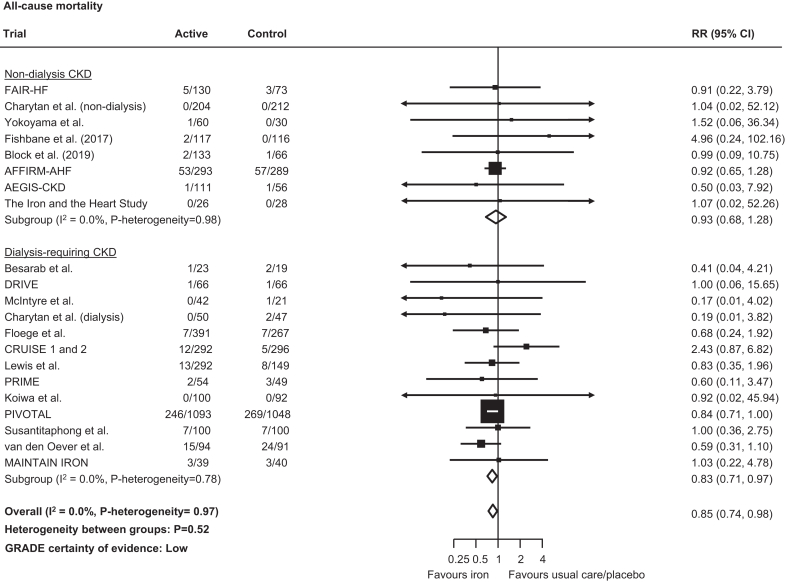
Effect of iron therapies versus usual care or placebo on all-cause mortality in people with CKD. CI, confidence interval; CKD, chronic kidney disease; GRADE, Grading of Recommendations, Assessment, Development and Evaluation; RR, relative risk.

**Figure 5:**
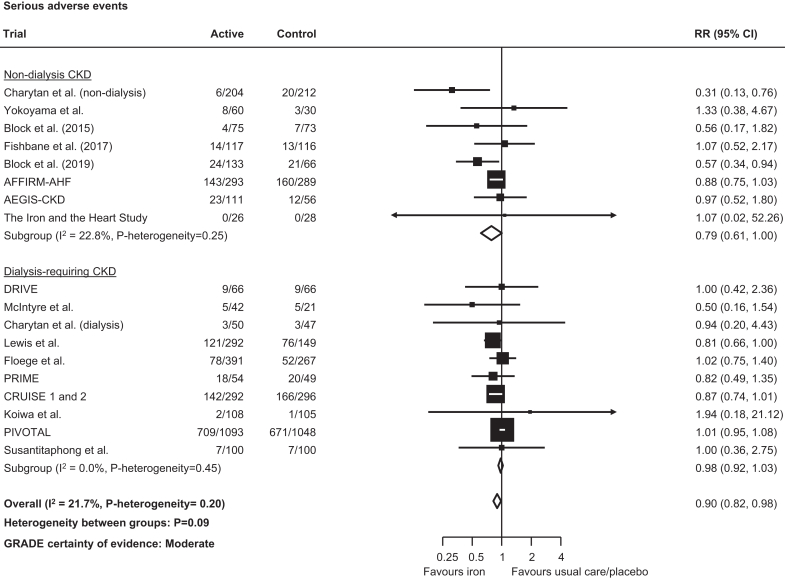
Effect of iron therapies vs. usual care or placebo on serious adverse events in people with CKD. CI, confidence interval; CKD, chronic kidney disease; GRADE, Grading of Recommendations, Assessment, Development and Evaluation; RR, relative risk.

Randomization to iron reduced the risk of all-cause mortality (765 events; RR: 0.85, 95% CI: 0.74–0.98; low certainty; Figure 4), driven largely again by the PIVOTAL trial in dialysis-requiring CKD, although no statistical evidence of heterogeneity was observed across dialysis- and nondialysis-requiring CKD subgroups (*P*-heterogeneity= 0.52). Fewer serious adverse events were observed with iron compared to usual care or placebo (2562 events, RR: 0.90, 95% CI: 0.82–0.98; moderate certainty; *P*-heterogeneity across CKD subgroups = 0.09; Figure 5). There were too few data to reliably assess effects on mortality and serious adverse events for high versus low-dose iron products (Supplementary Figure S4).

## Discussion

In this systematic review and meta-analysis, we observed that iron therapies reduced the risk of hospitalization for heart failure or cardiovascular death compared with placebo or usual care, with consistent effects in patients with dialysis- and nondialysis-requiring CKD. Although iron appeared to reduce the risk of MI, kidney failure, and all-cause mortality, the effects were imprecise or uncertain, often driven by a single trial not powered for these endpoints, and with very limited, if any, data on nondialysis-requiring CKD. For the comparisons of i.v. versus oral iron, newer versus older iron formulations, and higher versus lower oral iron doses, the lack of data precluded our ability to draw reliable inferences about their effects on cardiovascular events. Taken together, these data suggest that iron may yield beneficial cardiovascular effects in patients with CKD, particularly in those with heart failure; however, dedicated clinical trials of iron in patients with nondialysis-requiring CKD, with and without heart failure, are needed to guide clinical practice.

We should emphasize the fact that many of the trials included in our systematic review enrolled patients with heart failure and reduced ejection fraction; however, heart failure with preserved ejection fraction is the predominant form of heart failure in patients with CKD.^57^ To date, there are very limited data on the effects of i.v. iron on functional and clinical outcomes in heart failure with preserved ejection fraction, with data limited to a single trial of 39 participants.^58^

In contrast to trials testing the effects of iron administration in heart failure (where we examined results in the CKD subgroup), only a single dedicated CKD trial, PIVOTAL,^30^ was designed to evaluate the effects of iron on cardiovascular outcomes, though these outcomes were consistently identified as a priority for patients and their caregivers.^59^ Instead, CKD trials focused almost exclusively on correction of anemia and changes in ESA dosing. These trials were largely motivated by concerns about ESA safety in nondialysis-requiring CKD and economic considerations.^60^^,^^61^ The reduction in MI, driven largely by the PIVOTAL trial, raises the possibility that the cardiovascular benefits of iron in CKD may extend beyond reductions in heart failure and related events. However, without adequately powered cardiovascular outcome trials in patients with nondialysis CKD, its effects on MI or stroke remain uncertain.

Currently, there is a clear distinction in the recommendations of cardiology and nephrology clinical practice guidelines regarding the use of iron therapies. The European Society of Cardiology heart failure guidelines recommend i.v. iron in patients with heart failure with reduced ejection fraction who are iron deficient, to improve quality of life and to reduce the risk of hospitalization for heart failure.^6^ In contrast, the Kidney Disease Improving Global Outcomes 2012 guidelines for the management of anemia in CKD recommend a trial of iron (i.v. or oral) to increase hemoglobin without starting an ESA or to reduce the dose of ESA required to maintain hemoglobin concentrations and mitigate the need for red blood cell transfusions,^7^ with a similar approach outlined in the 2025 update. However, trials in patients with heart failure and reduced ejection fraction have indicated that the benefits of heart failure outcomes are independent of anemia.^5^

Our findings suggest that reconsidering the current recommendations on the use of iron in CKD might be appropriate and allow more patients to benefit from therapy with a favorable benefit-risk profile. A multinational study of almost 7000 patients across North America, Brazil, and Europe identified significant undertreatment of iron deficiency in patients with CKD, even among those with anemia.^1^

The lower incidence of serious adverse events with iron therapies versus usual care or placebo is reassuring, especially in patients with kidney failure, where there has previously been concerns about increased risk of infection and thrombosis.^62^ However, long-term safety data are lacking, because only 5 of the 17 studies evaluating serious adverse events had a follow-up duration of at least 12 months.^15^^,^^26^^,^^30^^,^^32^^,^^34^ In contrast, when comparing i.v. versus oral iron therapies, there was a marginally higher incidence of serious adverse events associated with i.v. iron. This was driven by 2 studies (FIND-CKD and REVOKE) which had longer follow-up periods (56 weeks and 24 months, respectively) than the other trials.^38^^,^^42^ These results emphasize the need for trials with repeated dosing of iron therapies to evaluate long-term efficacy and safety. Small trials, not specifically conducted in CKD, have suggested that newer iron formulations may be superior to older formulations with respect to safety^63^; however, we found no evidence that serious adverse events differed between i.v. versus oral iron and newer versus older iron formulations. Nevertheless, the overall safety profile of iron supports the feasibility of placebo-controlled outcome trials of iron therapies for CKD, designed to evaluate their effects on clinical outcomes.

Although the available evidence suggests a potential role of iron therapies in reducing risk of worsening heart failure in CKD, this comes with the caveat that almost all the data for this outcome were derived from trials of patients with heart failure with reduced ejection fraction. Thus, it is difficult to determine whether these effects are driven by the presence of heart failure with a reduced ejection fraction rather than by CKD. Although there were few available data on kidney outcomes, the finding that ferric citrate coordination complex (compared with usual care) reduced the need for dialysis in a single phase 2 trial raises the possibility that iron therapies may slow the progression of kidney disease, or ameliorate signs or symptoms of uremia (or heart failure) that might prompt dialysis initiation.^20^ These data, and the uncertainty they highlight, underscores the need for adequately powered randomized trials evaluating the effects of iron on functional status, health-related quality of life and clinical outcomes in patients with nondialysis-requiring CKD, with or without anemia. Indeed, the draft Kidney Disease Improving Global Outcomes 2025 Clinical Practice Guideline For Anemia in CKD highlights that “adequately powered pragmatic randomized controlled trials are needed to assess the benefits, harms, and costs of a proactive high dose i.v. iron regimen—in people with CKD not receiving dialysis.”

This study benefits from a prespecified, comprehensive, and systematic approach to the quantitative synthesis and interpretation of many randomized trials. The data were evaluated for multiple treatment comparisons. However, this study has some important limitations. This was a 2-stage tabular meta-analysis using mostly published data; thus, we were unable to assess treatment interactions across important subgroups such as those based on transferrin saturation, serum ferritin, or hemoglobin. Aside the large event-driven outcome trials, cardiovascular events were generally not independently adjudicated, which may be particularly relevant given the clinical overlap between heart failure and CKD progression. Data for some iron products, such as ferric derisomaltose, were available only for the primary outcomes. Limited data on MI, stroke, and CKD progression are reflected in imprecise treatment effect estimates and limited our ability to test for heterogeneity across the dialysis- and nondialysis-requiring CKD subgroups. Considering these limitations, our findings provide the most comprehensive assessment of the therapeutic landscape for iron therapies in CKD and underscore the need for randomized, placebo-controlled outcome trials in patients with CKD, especially those not on dialysis.

In summary, iron therapies may reduce the risk of hospitalization because of heart failure or cardiovascular death in patients with CKD. Randomized trials evaluating the effects of iron on clinical outcomes are warranted, especially in nondialysis patients with CKD with or without anemia.

## Disclosure

SVB reports consulting fees from Bayer, AstraZeneca, GSK, and Vifor Pharma; speaking fees from Bayer, AstraZeneca, Pfizer, and Vifor Pharma (all honoraria paid to his institution); and nonfinancial research support from Bayer. RPF is an employee of the Arbor Research Collaborative for Health, which receives global support for the ongoing DOPPS Programs (provided without restrictions on publications by a variety of funders; for details, see https://www.dopps.org/AboutUs/Supportcoworkers); has received research grants from Fresenius Medical Care; and consulting fees (paid to the employer) from AstraZeneca, Akebia, Novo Nordisk, Fresenius, Bayer, Boehringer, Novo Nordisk, and Akebia. GMC serves on the Board of Directors of Satellite Healthcare, a nonprofit dialysis provider; has served as the Chair or Co-Chair of Trial Steering Committees with Akebia, AstraZeneca, CSL Behring, Sanifit, and Vertex; served as an Advisor to Applaud, CloudCath, Durect, Eliaz Therapeutics, Miromatrix, Outset, Physiowave, Renibus, and Unicycive; and has served on Data Safety Monitoring Boards with Bayer, Mineralys, and ReCor. SDS has received research grants from Actelion, Alnylam, Amgen, AstraZeneca, Bellerophon, Bayer, BMS, Celladon, Cytokinetics, Eidos, Gilead, GSK, Ionis, Lilly, Mesoblast, MyoKardia, NIH/NHLBI, Neurotronik, Novartis, NovoNordisk, Respicardia, Sanofi Pasteur, Theracos, US2. AI; and has consulted for Abbott, Action, Akros, Alnylam, Amgen, Arena, AstraZeneca, Bayer, Boeringer-Ingelheim, BMS, Cardior, Cardurion, Corvia, Cytokinetics, Daiichi-Sankyo, GSK, Lilly, Merck, Myokardia, Novartis, Roche, Theracos, Quantum Genomics,Cardurion, Janssen, Cardiac Dimensions, Tenaya, Sanofi-Pasteur, Dinaqor, Tremeau, CellProThera, Moderna, American Regent, Sarepta, Lexicon, Anacardio, Akros, and Puretech Health. MV has received research grant support, served on advisory boards, or had speaker engagements with American Regent, Amgen, AstraZeneca, Bayer AG, Baxter Healthcare, BMS, Boehringer Ingelheim, Chiesi, Cytokinetics, Lexicon Pharmaceuticals, Merck, Novartis, Novo Nordisk, Pharmacosmos, Relypsa, Roche Diagnostics, Sanofi, and Tricog Health; and has participated in clinical trial committees for studies sponsored by AstraZeneca, Galmed, Novartis, Bayer AG, Occlutech, and Impulse Dynamics. VP has served as a consultant for AstraZeneca, Bayer Healthcare, Boehringer Ingelheim, Chinook Therapeutics, George Clinical, Gilead Sciences Inc., GlaxoSmithKline, Janssen Global Services LLC, Mundipharma, Novo Nordisk, Otsuka Pharmaceutical, Travere Therapeutics Inc., Tricida, and UptoDate. BLN received fees for travel support, advisory boards, publication support, scientific presentations, and steering committee roles from AstraZeneca, Alexion, Bayer, Boehringer Ingelheim, Cambridge Healthcare Research, Cornerstone Medical Education, the limbic, Janssen, Medscape, Menarini, Novo Nordisk, and Travere Therapeutics. All the other authors declared no conflicting interests.

## Supplementary Material

Supplementary File (PDF) **Figure S1.** Identification of eligible studies: PRISMA flow diagram. **Figure S2.** The effects of i.v. compared with oral iron on (A) heart failure hospitalization, (B) cardiovascular death, (C) myocardial infarction, (D) stroke, (E) any cause death, and (F) serious adverse events. **Figure S3.** The effects of newer versus older generation iron products on (A) heart failure hospitalization, (B) cardiovascular death, (C) myocardial infarction, (D) stroke, (E) any cause death, and (F) serious adverse events. **Figure S4.** The effect of high versus low-dose oral iron products on (A) all-cause mortality, and (B) serious adverse events. **Table S1.** Electronic search strategy. **Table S2.** Characteristics of included trials comparing i.v. versus oral iron therapies. **Table S3.** Characteristics of included trials comparing new versus older generation iron formulations. **Table S4.** Characteristics of included trials comparing higher vs. lower dose oral iron therapies. **Table S5.** Risk of bias assessment—iron versus usual care or placebo. **Table S6.** Risk of bias assessment—i.v. versus oral iron. **Table S7.** Risk of bias assessment—newer generation iron versus older generation iron formulations. **Table S8.** Risk of bias assessment—higher versus lower dose oral iron. **Table S9.** Changes in estimated glomerular filtration rate with iron therapy versus usual care or placebo. **Table S10.** Changes in estimated glomerular filtration rate with i.v. versus oral iron. **Table S11.** Changes in estimated glomerular filtration rate with higher versus lower dose oral iron. **Table S12.** Changes in proteinuria with iron therapy versus usual care or placebo. **Table S13.** Changes in proteinuria with newer versus older generation iron. **Table S14.** Changes in proteinuria with higher versus lower dose oral iron. **Table S15.** Changes in albuminuria with iron therapy versus usual care or placebo. **Table S16.** Changes in albuminuria with newer versus older generation iron.

## Footnotes

Supplementary File (PDF)
**Figure S1.** Identification of eligible studies: PRISMA flow diagram. **Figure S2.** The effects of i.v. compared with oral iron on (A) heart failure hospitalization, (B) cardiovascular death, (C) myocardial infarction, (D) stroke, (E) any cause death, and (F) serious adverse events. **Figure S3.** The effects of newer versus older generation iron products on (A) heart failure hospitalization, (B) cardiovascular death, (C) myocardial infarction, (D) stroke, (E) any cause death, and (F) serious adverse events. **Figure S4.** The effect of high versus low-dose oral iron products on (A) all-cause mortality, and (B) serious adverse events. **Table S1.** Electronic search strategy. **Table S2.** Characteristics of included trials comparing i.v. versus oral iron therapies. **Table S3.** Characteristics of included trials comparing new versus older generation iron formulations. **Table S4.** Characteristics of included trials comparing higher vs. lower dose oral iron therapies. **Table S5.** Risk of bias assessment—iron versus usual care or placebo. **Table S6.** Risk of bias assessment— i.v. versus oral iron. **Table S7.** Risk of bias assessment—newer generation iron versus older generation iron formulations. **Table S8.** Risk of bias assessment—higher versus lower dose oral iron. **Table S9.** Changes in estimated glomerular filtration rate with iron therapy versus usual care or placebo. **Table S10.** Changes in estimated glomerular filtration rate with i.v. versus oral iron. **Table S11.** Changes in estimated glomerular filtration rate with higher versus lower dose oral iron. **Table S12.** Changes in proteinuria with iron therapy versus usual care or placebo. **Table S13.** Changes in proteinuria with newer versus older generation iron. **Table S14.** Changes in proteinuria with higher versus lower dose oral iron. **Table S15.** Changes in albuminuria with iron therapy versus usual care or placebo. **Table S16.** Changes in albuminuria with newer versus older generation iron.
