# Supplementary material for: Systematic Review of the Effects of Iron on Cardiovascular, Kidney, and Safety Outcomes in Patients With CKD
Source: Kidney Int Rep. 2025 Jan 29;10(4):1037–49. doi: 10.1016/j.ekir.2025.01.029 (PMC12034885; doi:10.1016/j.ekir.2025.01.029)
Supplement: Supplementary File (PDF) — Figure S1. Identification of eligible studies: PRISMA flow diagram. Figure S2. The effects of i.v. compared with oral iron on (A) heart failure hospitalization, (B) cardiovascular death, (C) myocardial infarction, (D) stroke, (E) any cause death, and (F) serious adverse events. Figure S3. The effects of newer versus older generation iron products on (A) heart failure hospitalization, (B) cardiovascular death, (C) myocardial infarction, (D) stroke, (E) any cause death, and (F) serious adverse events. Figure S4. The effect of high versus low-dose oral iron products on (A) all-cause mortality, and (B) serious adverse events. Table S1. Electronic search strategy. Table S2. Characteristics of included trials comparing i.v. versus oral iron therapies. Table S3. Characteristics of included trials comparing new versus older generation iron formulations. Table S4. Characteristics of included trials comparing higher vs. lower dose oral iron therapies. Table S5. Risk of bias assessment—iron versus usual care or placebo. Table S6. Risk of bias assessment—i.v. versus oral iron. Table S7. Risk of bias assessment—newer generation iron versus older generation iron formulations. Table S8. Risk of bias assessment—higher versus lower dose oral iron. Table S9. Changes in estimated glomerular filtration rate with iron therapy versus usual care or placebo. Table S10. Changes in estimated glomerular filtration rate with i.v. versus oral iron. Table S11. Changes in estimated glomerular filtration rate with higher versus lower dose oral iron. Table S12. Changes in proteinuria with iron therapy versus usual care or placebo. Table S13. Changes in proteinuria with newer versus older generation iron. Table S14. Changes in proteinuria with higher versus lower dose oral iron. Table S15. Changes in albuminuria with iron therapy versus usual care or placebo. Table S16. Changes in albuminuria with newer versus older generation iron. [file mmc1.pdf]

Supplementary Appendix

Systematic Review of the Effects of Iron on Cardiovascular, Kidney and Safety Outcomes in Patients With Chronic Kidney Disease

Figure S1. Identification of eligible studies: PRISMA flow diagram.
Figure S2. The effects of intravenous compared to oral iron on (A) heart failure hospitalization, (B) cardiovascular death, (C) myocardial infarction, (D) stroke, (E) any cause death, and (F) serious adverse events.
Figure S3. The effects of newer vs. older generation iron products on (A) heart failure hospitalization, (B) cardiovascular death, (C) myocardial infarction, (D) stroke, (E) any cause death, and (F) serious adverse events.
Figure S4. The effect of high vs. low dose oral iron products on (A) all-cause mortality, and (B) serious adverse events.
Table S1. Electronic search strategy
Table S2. Characteristics of included trials comparing intravenous vs. oral iron therapies
Table S3. Characteristics of included trials comparing new vs. older generation iron formulations
Table S4. Characteristics of included trials comparing higher vs. lower dose oral iron therapies
Table S5. Risk of bias assessment – iron vs. usual care or placebo.
Table S6. Risk of bias assessment – intravenous vs. oral iron.
Table S7. Risk of bias assessment – newer generation iron vs. older generation iron formulations.
Table S8. Risk of bias assessment – higher vs. lower dose oral iron.
Table S9. Changes in eGFR with iron therapy vs. usual care or placebo
Table S10. Changes in eGFR with intravenous vs. oral iron
Table S11. Changes in eGFR with higher vs. lower dose oral iron
Table S12. Changes in proteinuria with iron therapy vs. usual care or placebo
Table S13. Changes in proteinuria with newer vs older generation iron
Table S14. Changes in proteinuria with higher vs. lower dose oral iron
Table S15. Changes in albuminuria with iron therapy vs. usual care or placebo
Table S16. Changes in albuminuria with newer vs older generation iron

Figure S1. Identification of eligible studies: PRISMA flow diagram.

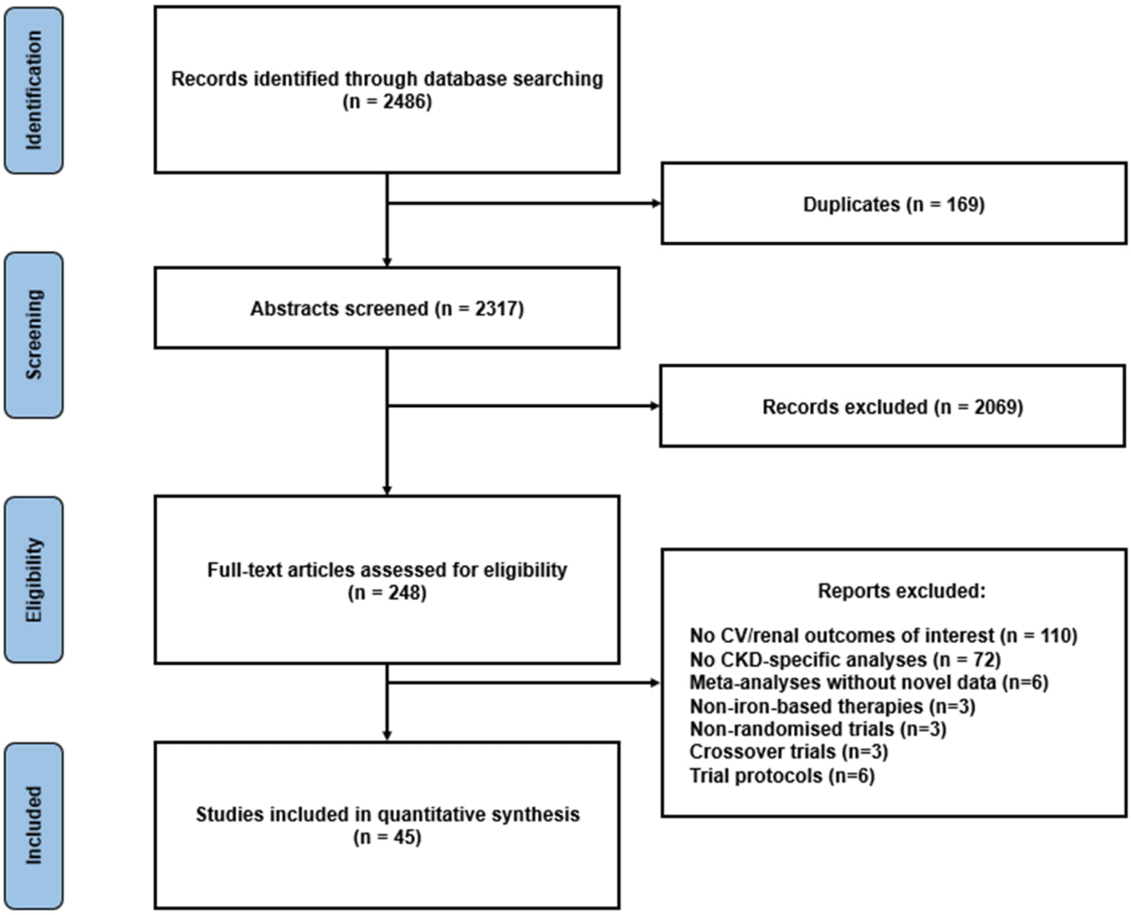

CV: cardiovascular; CKD: chronic kidney disease.

Figure S2. The effect of intravenous compared to oral iron therapies on (A) heart failure hospitalisation, (B) cardiovascular death, (C) myocardial infarction, (D) stroke, (E) all-cause mortality, (F) kidney failure requiring dialysis, and (G) serious adverse events

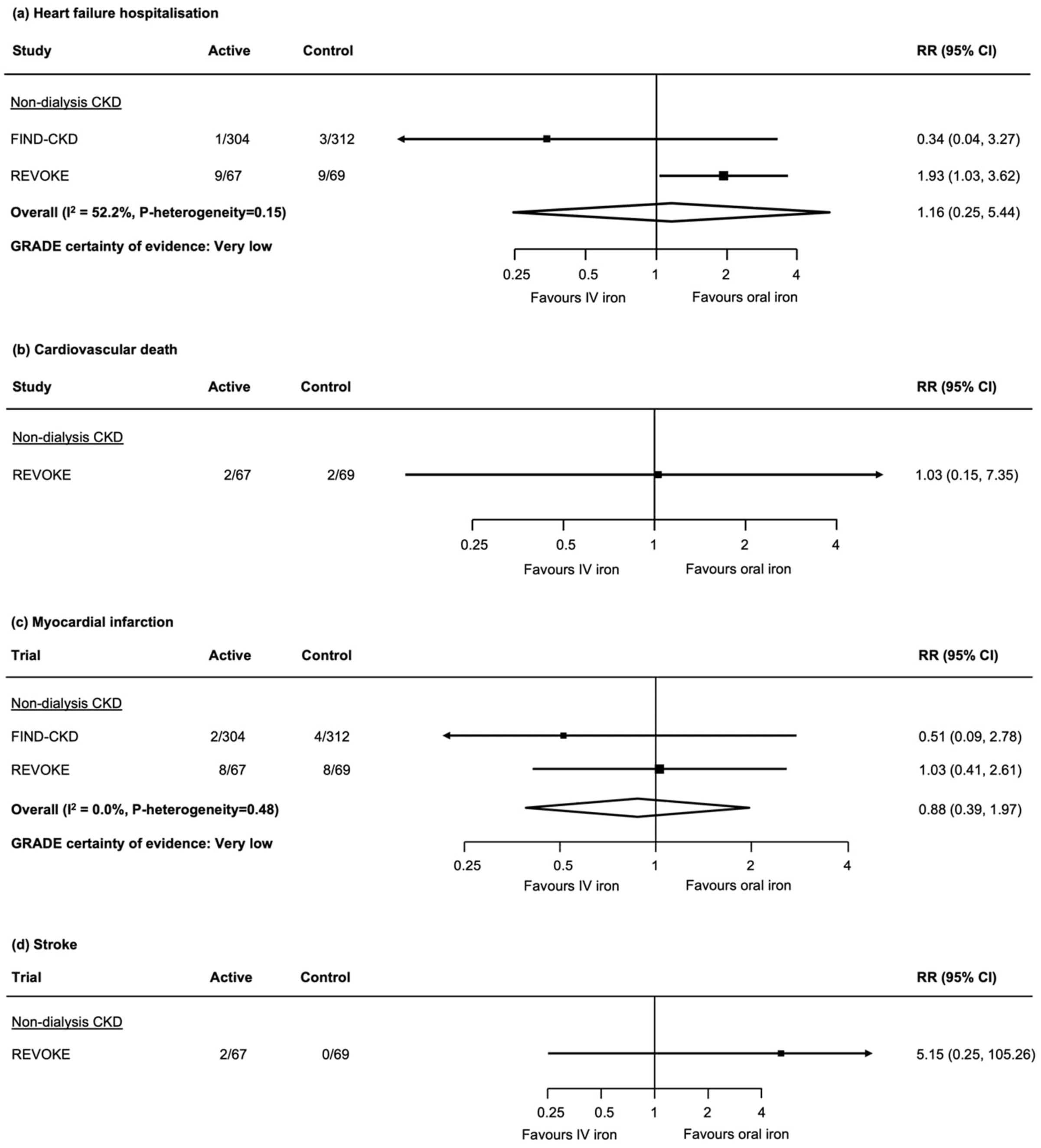

(e) All-cause mortality

Trial	Active	Control	RR (95% CI)
Non-dialysis CKD			
McMahon et al.	5/52	1/48	4.62 (0.56, 38.10)
FIND-CKD	10/304	15/312	0.68 (0.31, 1.50)
REVOKE	6/67	4/69	1.55 (0.44, 5.50)
Kalra et al. (2016)	3/228	0/117	3.61 (0.19, 69.25)
**Overall (I²= 26.3%, P-heterogeneity = 0.25)**			1.26 (0.55, 2.91)
**GRADE certainty of evidence: Very low**			

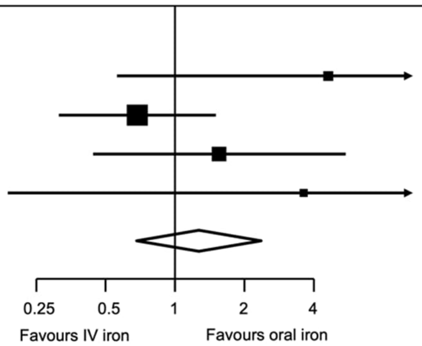

(F) Kidney failure requiring dialysis

Trial	Active	Control	RR (95% CI)
Non-dialysis CKD			
Van Wyck et al.	3/91	3/91	1.00 (0.21, 4.82)
McMahon et al.	1/52	3/48	0.31 (0.03, 2.86)
FIND-CKD	6/305	10/308	0.61 (0.22, 1.65)
REVOKE	6/67	7/69	0.89 (0.30, 2.64)
**Overall (I²= 0.0%, P-heterogeneity = 0.81)**			0.71 (0.38, 1.35)
**GRADE certainty of evidence: Very low**			

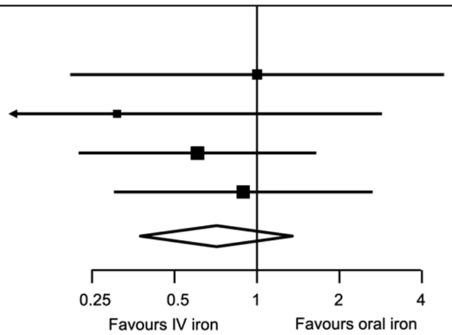

(g) Serious adverse events

Trial	Active	Control	RR (95% CI)
Non-dialysis CKD			
Aggarwal et al.	0/20	0/20	1.00 (0.02, 48.09)
Van Wyck et al.	1/91	0/91	3.00 (0.12, 72.69)
Agarwal et al. (2006)	3/44	0/45	7.16 (0.38, 134.62)
FIND-CKD	75/304	59/312	1.30 (0.96, 1.77)
REVOKE	37/67	40/69	1.18 (0.97, 1.45)
Pisani et al.	0/33	0/66	1.97 (0.04, 97.17)
Kalra et al. (2016)	3/228	1/117	1.54 (0.16, 14.64)
**Overall (I²= 0.0%, P-heterogeneity = 0.91)**			1.23 (1.04, 1.45)
**GRADE certainty of evidence: Low**			

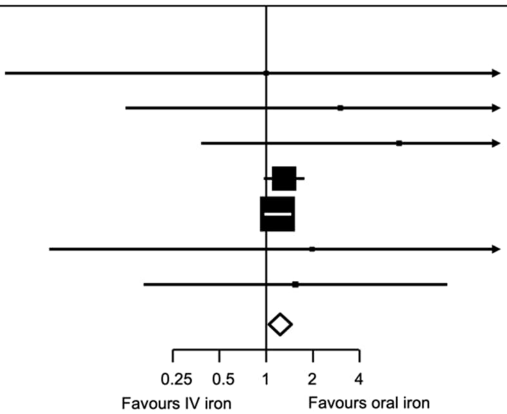

CKD: chronic kidney disease; RR: relative risk; CI: confidence interval.

Figure S3. The effect of newer vs. older generation iron formulations on (A) heart failure hospitalization, (B) cardiovascular death, (C) myocardial infarction, (D) stroke, (E) all-cause mortality, and (F) serious adverse events.

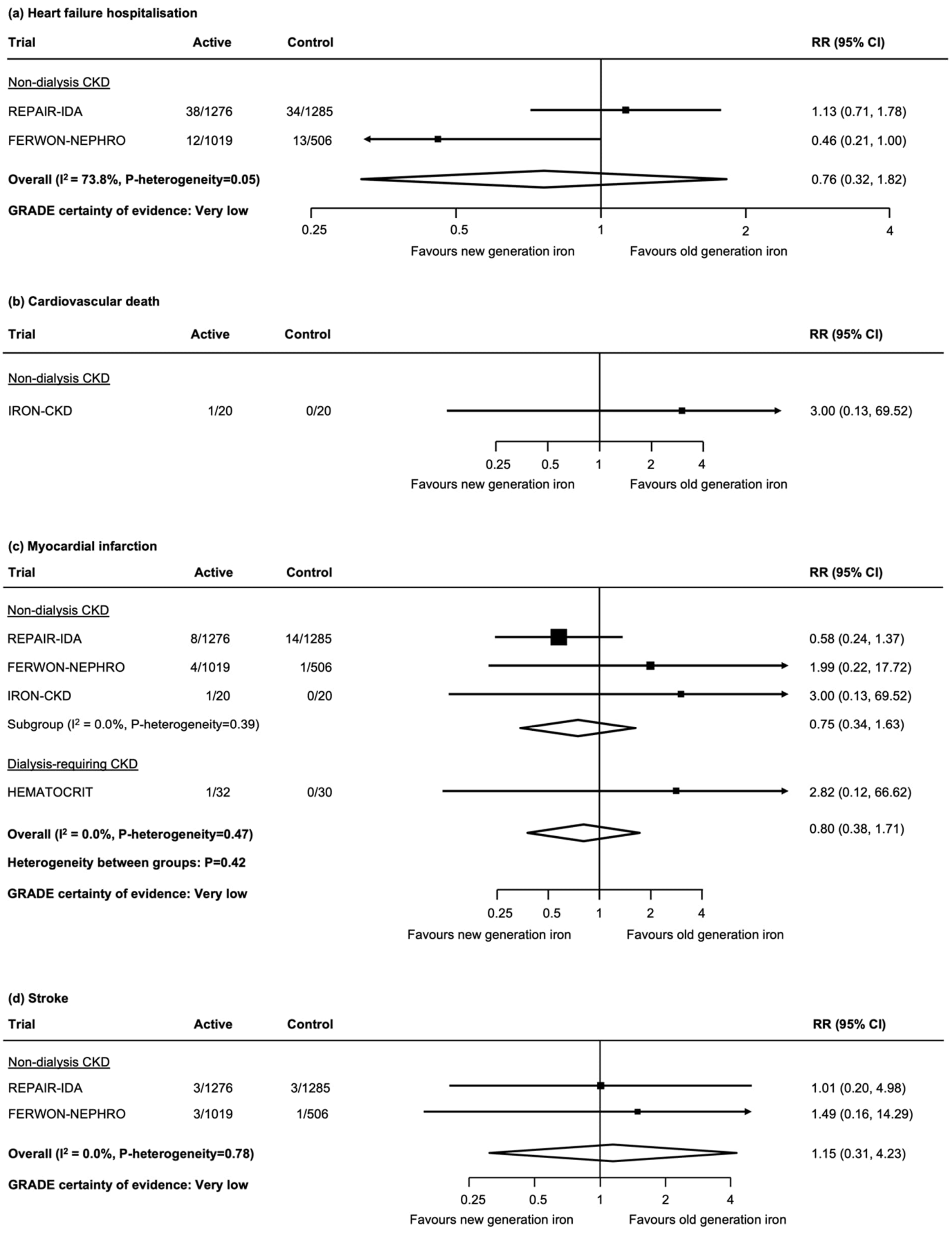

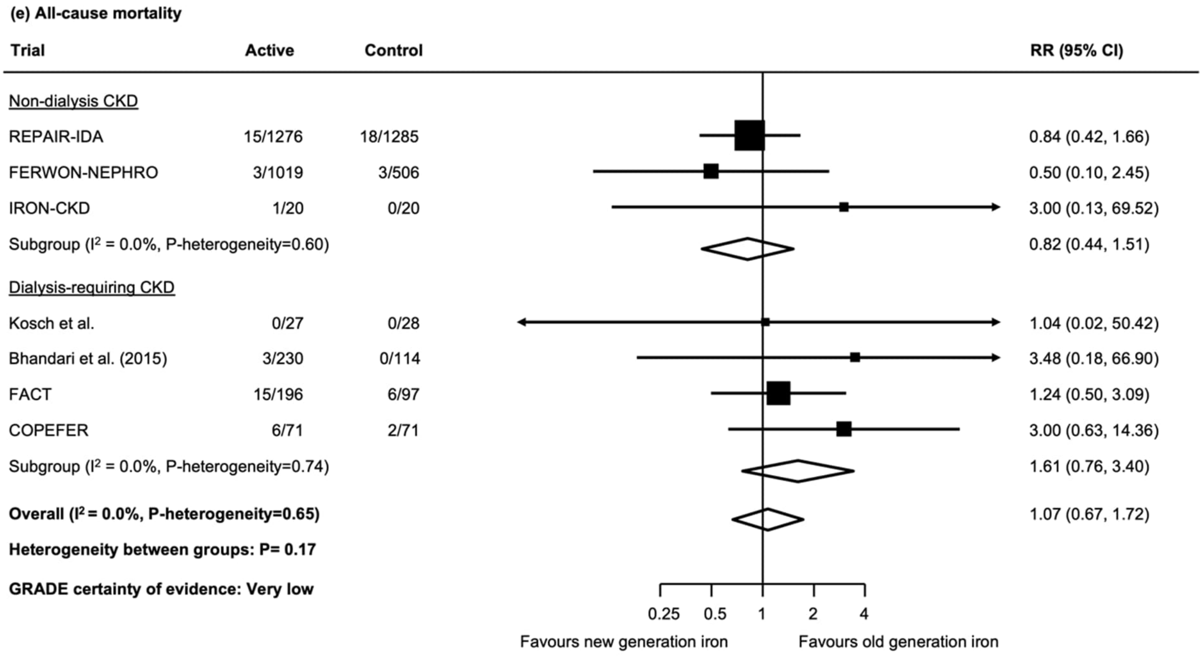

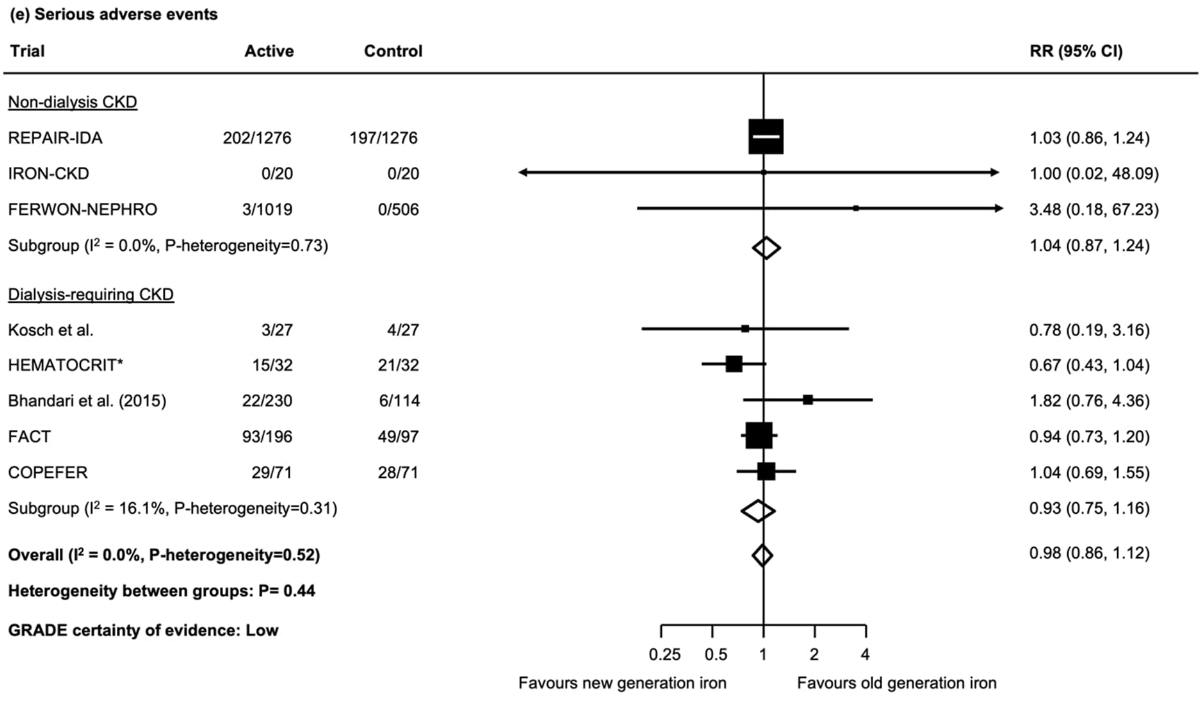

*Recurrent events analysis.

CKD: chronic kidney disease; RR: relative risk; CI: confidence interval.

Figure S4. The effect of high vs. low dose oral iron products on (A) all-cause mortality, and (B) serious adverse events.

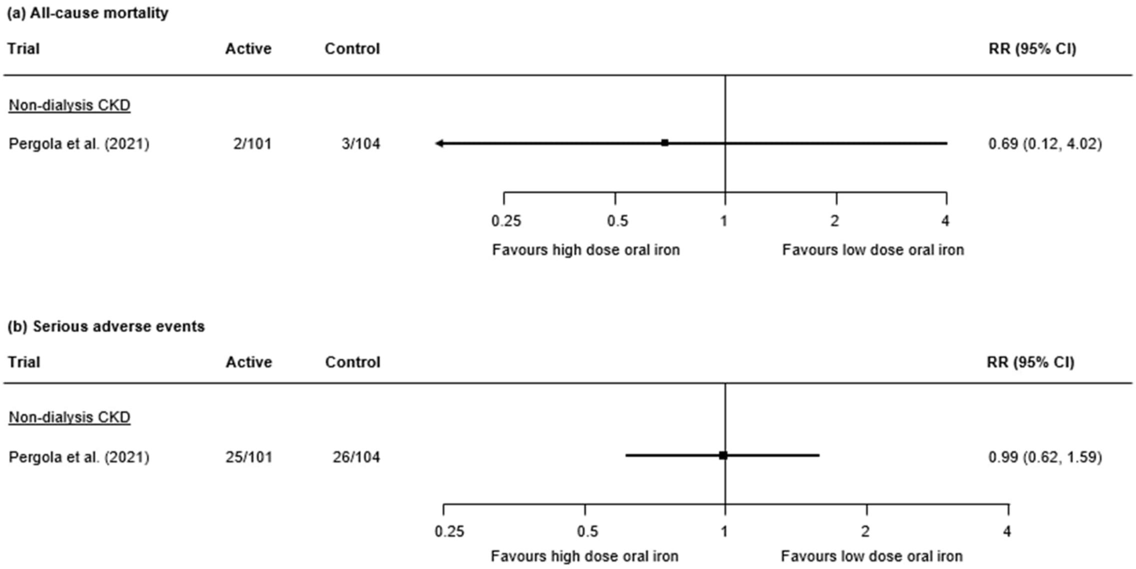

CKD: chronic kidney disease; RR: relative risk; CI: confidence interval.

Table S1. Electronic search strategy

**Database**	**Search terms**
Medline	1. exp Iron Compounds/ or exp Ferric Compounds/ or exp Ferrous Compounds/<br>2. exp Renal Insufficiency, Chronic/ or exp Renal insufficiency/ or Kidney Diseases/ or exp Kidney Failure, Chronic/ or exp Renal Dialysis/ or exp Dialysis/ or exp Renal Replacement Therapy/ or exp Heart failure/<br>3. exp Clinical Trial/ or exp Controlled Clinical Trial/ or exp Randomized Controlled Trial/ or exp Random Allocation/<br>4. 1 and 2 and 3<br>5. limit 4 to humans
Embase	1. ferric hydroxide sucrose/ or ferrous sulfate/ or ferric carboxymaltose/ or iron deficiency anemia/ or iron deficiency/ or iron saccharate/ or iron therapy/ or iron polymaltose/<br>2. exp chronic kidney disease/ or *chronic kidney disease/ or exp chronic kidney failure/ or kidney disease/ or peritoneal dialysis/ or renal replacement therapy/ or end stage renal disease/ or hemodialysis/ or heart failure with reduced ejection fraction/ or heart failure with preserved ejection fraction/ or *heart failure/ or cardiovascular mortality/<br>3. exp controlled clinical trial/ or exp clinical trial/ or exp controlled study/ or exp randomized controlled trial/<br>4. 1 and 2 and 3<br>5. limit 4 to humans
CENTRAL	1. MeSH descriptor: [Iron] explode all trees or<br>MeSH descriptor: [Iron Compounds] explode all trees or<br>MeSH descriptor: [Ferric Compounds] explode all trees or<br>MeSH descriptor: [Ferrous Compounds] explode all trees<br>2. MeSH descriptor: [Renal Insufficiency, Chronic] explode all trees or<br>MeSH descriptor: [Kidney Failure, Chronic] explode all trees or<br>MeSH descriptor: [Renal Insufficiency] explode all trees or<br>MeSH descriptor: [Kidney Diseases] explode all trees or<br>MeSH descriptor: [Renal Dialysis] explode all trees or<br>MeSH descriptor: [Renal Replacement Therapy] explode all trees or<br>MeSH descriptor: [Peritoneal Dialysis] explode all trees or<br>MeSH descriptor: [Heart Failure] explode all trees<br>3. 1 and 2

Table S2. Characteristics of included trials comparing intravenous vs. oral iron therapies.

Trial	Year	No. of Participants with CKD / Total Study Participants	Duration of Follow-up	Primary Population	Intervention	Comparator	Mean Age	Proportion Female (%)	Primary Outcome
Aggarwal et al.[40]	2003	40 / 40	3 months	Non-dialysis CKD	IV iron dextran	PO ferrous sulfate	NR	27.5	Change in Hb, packed cell volume and reticulocyte count
VanWyck et al.[45]	2005	182 / 182	56 days	Non-dialysis CKD	IV iron sucrose	PO ferrous sulfate	63.1	67.7	Proportion of patients with Hb rise ≥1.0 g/dL
Agarwal et al.[39]	2006	75 / 75	43 days	Non-dialysis CKD	IV sodium ferric gluconate complex	PO ferrous sulfate	63.8	58.7	Change in Hb
McMahon et al.[43]	2010	85 / 85	12 months	Non-dialysis CKD	IV iron sucrose	PO ferrous sulfate	69.0	27.0	Change in Hb
FIND-CKD[42]	2014	626 / 626	56 weeks	Non-dialysis CKD	IV ferric carboxymaltose	PO ferrous sulfate	69.1	62.2	Time to initiation of ESA, other iron therapy or blood transfusion or 2 consecutive values Hb<10g/dL
REVOKE[38]	2015	136 / 136	24 months	Non-dialysis CKD	IV iron sucrose	PO ferrous sulfate	65.5	23.5	Slope of measured GFR change
Pisani et al.[44]	2015	99 / 99	3 months	Non-dialysis CKD	IV iron gluconate	PO pyrophosphate liposomal iron	51.3	71.7	Change in Hb
Kalra et al.[41]	2016	351 / 351	4 weeks	Non-dialysis CKD	IV ferric derisomaltose	PO ferrous sulfate	57.7	55.6	Change in Hb

CKD: Chronic kidney disease; IV: intravenous; PO: per oral; Hb: haemoglobin; ESA: erythropoietin stimulating agent; GFR: glomerular filtration rate.

Table S3. Characteristics of included trials comparing newer vs. older iron formulations.

Trial	Year	No. of Participants with CKD	Duration of Follow-up	Primary Population	Intervention	Comparator	Mean Age	Proportion Female (%)	Primary Outcome
Kosch et al.[52]	2001	59 / 59	6 months	Hemodialysis	IV iron sucrose	IV iron gluconate	NR	NR	Change in Hb
Agarwal et al.[46]	2011	62 / 62	36 days	Non-dialysis CKD	IV ferric gluconate	IV iron sucrose	65.3	58.1	Urine protein to creatinine ratio
HEMATOCRIT[47]	2012	62 / 62	6 months	Peritoneal dialysis	PO heme iron polypeptide	PO ferrous sulfate	59.5	57.4	Transferrin saturation
REPAIR-IDA[54]	2014	2584 / 2584	56 days	Non-dialysis CKD	IV ferric carboxymaltose	IV iron sucrose	67.3	63.6	Change in Hb
Bhandari et al.[49]	2015	351 / 351	6 weeks	Hemodialysis	IV ferric derisomaltose	IV iron sucrose	59.9	34.2	Proportion of patients with Hb 9.5-12.5 g/dL at 6 weeks
FACT[53]	2019	293 / 293	11 months	Hemodialysis	IV ferumoxytol	IV iron sucrose	58.7	41.6	Change in Hb
COPEFER[50]	2021	142 / 142	40 weeks	Hemodialysis	IV ferric carboxymaltose	IV iron sucrose	58.5	28.9	Change in Hb
IRON-CKD[51]	2021	40 / 40	3 months	Non-dialysis CKD	IV ferric derisomaltose	IV iron sucrose and iron dextran	58.8	42.5	Oxidative stress (measured by thiobarbituric acid reactive substances and labile plasma iron) and inflammation (measured by interleukin and CRP levels)
FERWON-NEPHRO[48]	2021	1538 / 1538	8 weeks	Non-dialysis CKD	IV ferric derisomaltose	IV iron sucrose	68.6	62.5	Change in Hb and serious or severe hypersensitivity reactions

CKD: Chronic kidney disease; IV: intravenous; PO: per oral; Hb: haemoglobin; CRP: C-reactive protein.

Table S4. Characteristics of included trials comparing higher vs. lower dose oral iron therapies

Trial	Year	No. of Participants with CKD	Duration of Follow-up	Primary Population	Intervention	Comparator	Mean Age	Proportion Female (%)	Primary Outcome
Pergola et al.[55]	2021	206 / 206	48 weeks	Non-dialysis CKD	PO ferric citrate	PO ferric citrate	69.5	64.3	Change in Hb
Sood et al.[56]	2023	80 / 80	12 weeks	Non-dialysis CKD	PO ferric citrate	PO ferric citrate	50.0	30.0	Change in transferrin saturation

CKD: Chronic kidney disease; IV: intravenous; PO: per oral; Hb: haemoglobin.

Table S5. Risk of bias assessment – iron vs. usual care or placebo.

Trial	Random sequence generation	Allocation concealment	Blinding of participants and personnel	Blinding of outcome assessment	Incomplete outcome data	Selective Reporting	Other bias
Besarab et al.[18]	?	?	+	+	+	+	+
DRIVE[23]	+	+	+	?	+	?	+
FAIR-HF[12]	+	+	+	+	+	+	+
McIntyre et al.[31]	+	+	+	?	+	+	+
Charytan et al.[22]	+	+	+	+	+	+	+
Yokoyama et al.[36]	+	+	+	+	+	+	+
Block et al.[21]	+	+	+	+	+	+	+
CONFIRM-HF[25]	+	+	+	+	+	+	+
CRUISE 1 and 2[25]	+	+	?	+	+	+	+
PRIME[27]	+	+	+	+	+	+	+
Floege et al.[26]	+	+	+	+	+	+	+
Lewis et al.[34]	+	+	?	+	+	+	+
Fishbane et al.[24]	+	+	+	+	+	+	+
Koiwa et al.[29]	+	+	+	?	+	+	+
Iguchi et al.[28]	-	?	-	+	-	?	?
Block et al.[20]	+	+	?	?	+	+	+
PIVOTAL[30]	+	+	+	+	+	+	+
AFFIRM-AHF[15]	+	+	+	+	+	+	+
van den Oever et al.[35]	+	+	-	+	?	+	?

Susantitaphong et al.[33]	+	+	?	+	+	+	+
AEGIS-CKD[32]	+	+	+	+	+	+	+
ZununiVahed et al.[37]	?	+	+	+	+	+	+
The Iron and the Heart Study[19]	+	+	+	+	+	+	+
IRONMAN[13]	+	+	+	+	+	+	+
HEART-FID[14]	+	+	+	+	+	+	+
MAINTAIN-IRON[17]	+	+	+	+	+	+	+

Key + Low risk of bias - High risk of bias ? Unclear risk of bias

Table S6. Risk of bias assessment – intravenous vs. oral iron.

Trial	Random sequence generation	Allocation concealment	Blinding of participants and personnel	Blinding of outcome assessment	Incomplete outcome data	Selective Reporting	Other bias
Aggarwal et al.[40]	?	?	-	?	+	+	?
VanWyck et al.[45]	?	?	?	?	+	+	?
Agarwal et al.[39]	+	+	+	?	+	-	?
McMahon et al.[43]	+	+	-	+	?	+	?
FIND-CKD[42]	+	+	+	+	+	+	+
REVOKE[38]	+	+	-	?	+	+	?
Pisani et al.[44]	+	+	?	-	+	+	?
Kalra et al.[41]	+	+	?	-	+	+	?

Key + Low risk of bias - High risk of bias ? Unclear risk of bias

Table S7. Risk of bias assessment – newer generation iron vs. older generation iron formulations.

Trial	Random sequence generation	Allocation concealment	Blinding of participants and personnel	Blinding of outcome assessment	Incomplete outcome data	Selective Reporting	Other bias
Kosch et al.[52]	?	?	?	-	+	+	?
Agarwal et al.[46]	+	+	+	+	+	-	?
HEMATOCRIT[47]	+	+	+	?	+	+	?
REPAIR-IDA[54]	+	+	+	+	+	+	+

Bhandari et al.[49]	+	+	+	?	+	+	+
FACT[53]	+	+	+	?	+	+	+
COPEFER[50]	+	+	+	+	?	+	+
IRON-CKD[51]	+	+	-	?	+	+	?
FERWON-NEPHRO[48]	+	+	+	+	+	+	+

Key + Low risk of bias - High risk of bias ? Unclear risk of bias

Table S8. Risk of bias assessment – higher vs. lower dose oral iron.

**Trial**	**Random sequence generation**	**Allocation concealment**	**Blinding of participants and personnel**	**Blinding of outcome assessment**	**Incomplete outcome data**	**Selective Reporting**	**Other bias**
Pergola et al.[55]	?	?	?	?	+	+	?
Sood et al.[56]	+	+	?	+	+	+	+

Key + Low risk of bias - High risk of bias ? Unclear risk of bias

Table S9. Changes in eGFR (mL/min/1.73m^2) with iron therapy vs. usual care or placebo.

Trial	Iron		Usual care or placebo	
	Mean (SD), baseline	Mean (SD), follow-up	Mean (SD), baseline	Mean (SD), follow-up
The Iron and the Heart Study[19]	33.2 (9.3)	32.1 (9.5)	29.1 (9.9)	28.2 (8.6)
Iguchi et al.[28]	24.4 (9.8)	25.0 (11.2)	31.3 (10.1)	32.1 (11.0)
Yokoyama et al.[36]	8.6 (3.9)	7.9 (4.3)	9.8 (8.2)	9.0 (7.3)
FAIR-HF[12]	7.1 ± 1.24 (SE)		5.56 ± 1.74 (SE)	

SD: standard deviation; SE: standard error.

Table S10. Changes in eGFR (mL/min/1.73m^2) with intravenous vs. oral iron.

Trial	Intravenous iron		Oral iron	
	Mean (SD), baseline	Mean (SD), follow-up	Mean (SD), baseline	Mean (SD), follow-up
REVOKE[38]	34.3 (10.2)	NR	34.7 (10)	NR
	-4 per year (NR)		-3.6 per year (NR)	
	Between group differences: -0.35 (95% CI -2.9 to 2.3)			
Pisani et al.[44]	31.8 (12.9)	27.9 (7.8)	25.9 (11.4)	25.1 (12.7)
FIND-CKD[42]	32.0 (1.1)	33.2 (1.4)	33.2 (0.8)	33.7 (1.0)
	-0.6 (0.8)		-1.1 (0.6)	
McMahon et al.[43]	25.0 (8.0)	23.0 (8.0)	26.0 (11.0)	22.0 (10.0)
VanWyck et al.[45]	30.4 (NR)	NR	28.5 (NR)	NR
	-1.45 (95% CI -2.67 to -0.2)		-4.4 (95% CI -6.29 to -2.5)	

SD: standard deviation; CI: confidence interval. NR: not reported.

Table S11. Changes in eGFR (mL/min/1.73m^2) with higher vs. lower dose oral iron.

Trial	Higher dose oral iron		Lower dose oral iron	
	Mean (SD), baseline	Mean (SD), follow-up	Mean (SD), baseline	Mean (SD), follow-up
Sood et al.[56]	33.8 (11.1)	34.3 (16.3)	39.5 (12.4)	38.6 (15.7)
Pergola et al.[55]	34.5 (11.6)	NR	32.8 (10.1)	NR
	-2.20 (95% CI -4.24 to -0.15)		-1.85 (95% CI -3.78 to -0.08)	

SD: standard deviation; CI: confidence interval; NR: not reported.

Table S12. Changes in proteinuria (mg/g) with iron therapy vs. usual care or placebo.

Trial	Iron	Usual care or placebo

	Mean (SD), baseline (mg/g)	Mean (SD), follow-up (mg/g)	Mean (SD), baseline (mg/g)	Mean (SD), follow-up (mg/g)
The Iron and the Heart Study[19]	458.8 (524.2)	513.6 (815.9)	996.3 (1456.8)	618.8 (902.6)

SD: standard deviation.

Table S13. Changes in proteinuria with newer vs older generation iron.

Trial	On ACEI / ARB		No ACEI/ARB	
Agarwal et al.[46]	Newer generation iron (% change in UPCR from baseline)	Older generation iron (% change in UPCR from baseline)	Newer generation iron (% change in UPCR from baseline)	Older generation iron (% change in UPCR from baseline)
	NR	NR	NR	NR
	Iron sucrose (older generation) produced 78% higher UPCR compared to ferric gluconate (newer generation)		Iron sucrose (older generation) produced 40.9% higher UPCR compared to ferric gluconate (newer generation)	

NR: not reported.

Table S14. Changes in proteinuria (mg/g) with higher vs. lower dose oral iron.

Trial	Higher dose		Lower dose	
	Median (IQR), baseline (mg/g)	Median (IQR), follow-up (mg/g)	Median (IQR), baseline (mg/g)	Median (IQR), follow-up (mg/g)
Sood et al.[56]	0.54 (0.17-1.74)	0.37 (0.24-1.13)	0.30 (0.13-0.95)	0.46 (0.205-1.00)

IQR: interquartile range.

Table S15. Changes in albuminuria (mg/g) with iron therapy vs. usual care or placebo.

Trial	Iron		Usual care or placebo	
	Mean (SD), baseline (mg/g)	Mean (SD), follow-up (mg/g)	Mean (SD), baseline (mg/g)	Mean (SD), follow-up (mg/g)
The Iron and the Heart Study[19]	237.8 (353.6)	437.6 (633.8)	838.0 (1603.6)	375.7 (395.1)

Table S16. Changes in albuminuria with newer vs older generation iron.

Trial	On ACEI / ARB		No ACEI/ARB	
Agarwal et al.[46]	Newer generation iron (% change in UACR from baseline)	Older generation iron (% change in UACR from baseline)	Newer generation iron (% change in UACR from baseline)	Older generation iron (% change in UACR from baseline)
	NR	NR	NR	NR
	Iron sucrose (older generation) produced 135% higher UACR		NR	

	compared to ferric gluconate (newer generation)	

NR: not reported.
